# Adopting the Mediterranean Diet: Motivational and Socio-Cognitive Processes in Young Adults

**DOI:** 10.3390/healthcare14040509

**Published:** 2026-02-17

**Authors:** Marika Gentile, Luigi Tinella, Fabio Alivernini, Sara Manganelli, Fabio Lucidi, Laura Girelli

**Affiliations:** 1 Department of Humanities, Philosophy and Education, University of Salerno, 84084 Fisciano, Italy; marika.gentile00@libero.it (M.G.);; 2 Department of Developmental and Social Psychology, Sapienza University of Rome, Via dei Marsi 78, 00185 Rome, Italy

**Keywords:** mediterranean diet, motivation, intention, perceived behavioral control, young adults

## Abstract

Background/Objectives: Adherence to the Mediterranean Diet (MD) is associated with substantial physical and psychological health benefits, yet its adoption remains challenging during emerging adulthood. Although previous research has identified motivational and socio-cognitive determinants of specific eating behaviors, less is known about the psychological processes underlying adherence to the MD as a whole in youth. The present study examined the role of motivational factors derived from Self-Determination Theory (SDT) and key socio-cognitive variables from the Theory of Planned Behavior (TPB) in explaining adherence to the MD in young adults. Methods: A sample of 365 young adults (50.1% female; mean age = 21.74 years, SD = 5.86) completed an online questionnaire assessing motivational regulations, perceived behavioral control (PBC), behavioral intention, and adherence to the MD. Results: Structural equation modeling showed an excellent fit of the hypothesized model. Adherence to the MD was directly associated with behavioral intention (β = 0.18, *p* < 0.05), PBC (β = 0.24, *p* < 0.01), and intrinsic motivation (β = 0.22, *p* < 0.05). Behavioral intention was positively associated with PBC (β = 0.48, *p* < 0.001) and intrinsic motivation (β = 0.21, *p* < 0.05) and negatively associated with amotivation (β = −0.23, *p* < 0.05). Integrated regulation showed a significant indirect effect on intention via PBC. The model accounted for 40% of the variance in intention and 16% of the variance in adherence. Conclusions: The results suggest that interventions targeting this population should strengthen dietary intentions, enhance PBC, and foster autonomous motivation. The integrated model provides a useful framework for designing healthcare and public health interventions aimed at promoting healthy eating during emerging adulthood.

## 1. Introduction

Obesity represents a global public health emergency. According to the World Health Organization (WHO) [1], in 2022, 43% of adults aged 18 and over were overweight, while 16% suffered from obesity. Within this context, emerging adults (18–25 years) show an even higher prevalence of overweight and obesity. Indeed, more than 40% of young adults in this age range appear to struggle with excess body weight [2]. During this stage of life, obesity can contribute to low self-esteem, body dissatisfaction, depression, and anxiety, effects that are often intensified by weight-related social stigma [3]. Moreover, obesity in young adults increases the risk of developing chronic diseases such as type 2 diabetes and cardiovascular conditions [2]. The severity of obesity also appears to be directly associated with psychological distress in young adults: Kundi et al. [4] found that young adults with class III obesity showed 1.4 times higher psychological distress than their normal-weight peers, especially among women and Caucasian individuals. These findings highlight the need to develop an integrated approach to the issue. Current interventions, according to Alves et al. [5], are mainly focused on weight loss and often neglect the emotional and social dimensions of obesity within this age group.

One well-established approach to healthy eating is adherence to the Mediterranean Diet (MD). The typical nutrients of the MD (unsaturated fats, fiber, antioxidants, lean proteins) help reduce inflammation, oxidative stress, and cardiovascular risk. The MD proves to be sustainable, effective, and consistent with international nutritional recommendations. Estruch et al. [6] demonstrated that following a MD rich in olive oil and nuts reduces the risk of cardiovascular diseases by up to 30%. A study by Sofi et al. [7] shows that greater adherence to this dietary pattern is associated with lower mortality and a reduced risk of developing diseases such as cancer, Alzheimer’s, and Parkinson’s. According to Esposito et al. [8] and Salas-Salvadò et al. [9], the MD lowers the prevalence of type 2 diabetes and metabolic syndrome, improving insulin sensitivity and lipid profile. Trichopoulou et al. [10] also reported a significant reduction in overall mortality among those who follow the Mediterranean dietary pattern. Several studies show that the MD helps to improve cardiovascular health and reduce the risk of chronic diseases. Furthermore, numerous studies show that the MD not only improves physical health but also mental health and good psychological balance. Research by Lassale et al. [11] and Sánchez-Villegas et al. [12,13] indicates that high adherence to the MD is associated with a lower risk of depression, while Jacka et al. [14] demonstrate that adherence to this dietary pattern is linked to greater effectiveness in treating depressive disorders. Healthy fats, antioxidants, vitamins, and omega-3s—all key nutrients of this diet—support neurochemical balance.

Those involved in obesity prevention know that asking young adults to “go on a diet” is rarely effective. Encouraging adherence to a dietary regimen alone is often insufficient. Many young people discontinue diets before achieving lasting results. Effective nutritional interventions for young adults should therefore target the factors that best predict healthy eating behaviors and adherence to the Mediterranean diet.

Many studies have shown that socio-cognitive factors such as intention and perceived behavioral control (PBC) influence the adoption of healthy eating behaviors [15,16,17]. According to the Theory of Planned Behavior (TPB) [18], behavior is primarily determined by behavioral intention, which in turn is influenced by PBC [2,19]. PBC refers to an individual’s perception of their ability to perform a behavior while considering obstacles and available resources. Perceived control over food choices, such as feeling capable of preparing healthy meals, plays an important role in shaping intentions to eat healthily.

Some studies have also focused on the motivational aspect of eating behavior [20,21]. According to Self-Determination Theory (SDT) [22], motivations are placed along a continuum of self-determination, ranging from less autonomous to more autonomous forms: amotivation is associated with a specific regulatory style, namely non-regulation, which occurs when individuals do not act or act without a specific goal, perceiving the reasons for the action as irrelevant. Extrinsic motivation develops through three regulatory styles. External regulation refers to behavior driven by external factors such as rewards or punishments, which is therefore difficult to maintain without external incentives [22]. Introjected regulation is characterized by internal pressures, such as the pursuit of self-esteem or the avoidance of guilt [18]. Identified regulation occurs when individuals attribute personal value to the goal and perceive the activity as meaningful, acting with a greater sense of choice [23]. Intrinsic motivation represents the most autonomous form of regulation. It is based on an internal locus of causality, whereby individuals engage in an activity for the pleasure and satisfaction it provides, without the need for external rewards or pressures [23,24,25]. For example, Caso et al. [21] showed that motivational processes can effectively promote changes in eating behavior.

The TPB and SDT represent particularly suitable frameworks for understanding adherence to the MD during emerging adulthood. This developmental stage, which lies between late adolescence and early adulthood, is characterized by profound changes, including identity exploration, increasing autonomy, changes in social norms, and transitions in living arrangements [26]. During this period, young adults are often required to make independent decisions regarding food purchasing, meal preparation, and daily routines, frequently for the first time. In this context, PBC becomes a crucial factor in determining the ability to acquire and maintain healthy eating habits, while motivational regulation helps explain why some individuals adhere to the MD out of a growing sense of identity, autonomy, and personal well-being rather than external pressure.

Previous studies integrating TPB and SDT have provided empirical support for this framework. Canova et al. [20] examined the pathway from motivation to intention and subsequent adherence to the MD in adult samples, showing that motivational processes influence dietary behavior through socio-cognitive mechanisms. Similarly, Caso et al. [21] conducted a randomized controlled trial in an adult sample, demonstrating the effectiveness of an intervention based on the integration of the two theories in improving adherence to the MD. These findings confirm the central role of PBC and autonomous motivation in explaining health-related behaviors. However, both studies were conducted on adult samples. In a younger population, Girelli et al. [15] found that more autonomous forms of motivation predict intention and specific healthy eating behaviors, with PBC acting as a mediator, in a sample of high-school students and with reference to specific dietary behaviors (fruit and vegetable, breakfast, and snack consumption).

Building on this body of literature, research integrating TPB and SDT has increasingly highlighted PBC and behavioral intention as key socio-cognitive processes underlying healthy eating behaviors [27,28,29,30]. Emphasizing these constructs allows for a more parsimonious framework that can be effectively integrated with motivational dimensions derived from SDT.

To our knowledge, no previous study has tested an integrated TPB-SDT model of MD adherence, operationalized through selected TPB constructs, in a sample of emerging adults, using a detailed MD index. Therefore, the present study aims to examine the joint contribution of motivational factors derived from SDT and selected socio-cognitive constructs from the TPB—namely PBC and behavioral intention—in explaining adherence to the MD. By focusing on a sample of young adults, the study addresses a developmental stage that has been relatively underexplored in this line of research. The findings may provide useful insights into the design of nutrition and health promotion interventions in real-world settings. For example, in university health services, this model may inform educational and psychological support programs aimed at helping students develop eating habits consistent with the MD and aligned with their values and life goals. In primary care, nutritional counseling programs may benefit from strengthening both PBC and more autonomous forms of motivation. Finally, the model may also be applied in public health campaigns targeting young adults, shifting the focus from prescriptive messages toward approaches that foster autonomy, motivation, and a sense of control, thereby supporting sustained adherence to the MD over time.

## 2. Materials and Methods

### 2.1. Participants

The sample consisted primarily of undergraduate and master’s students from different academic disciplines at the University of Salerno. A small proportion of young adults not currently enrolled at the University of Salerno, or not enrolled at any university at all, were also included. Overall, the sample represents a convenience sample of young adults. Participants were aged between 18 and 30 years (M = 21.7, SD = 5.86), and the gender distribution was balanced (50.1% women). Eligibility criteria included being between 18 and 30 years of age and being able to complete an online questionnaire in Italian.

### 2.2. Procedure

Data were collected between October and November 2024, using an online questionnaire administered via Google Forms. Participants were recruited primarily during university lectures through a QR code displayed in the classroom, as well as through peer sharing via messaging apps. Data collection took place at the University of Salerno, located in the Campania region (Italy).

Participation was voluntary and anonymous. All participants provided informed consent prior to completing the questionnaire. The questionnaire collected basic sociodemographic information, including age, gender, place of residence, and university affiliation.

### 2.3. Instruments

Motivation was assessed using an adapted version of the Eating Behavior Regulation Scale (REBS) [31]. The original REBS consists of 24 items rated on a 7-point Likert scale ranging from 1 (not at all true) to 7 (completely true), in response to the question: “Why do you eat healthy?”. The items assessed six types of eating behavior regulation: intrinsic motivation (e.g., “Because it is fun”), integrated regulation (e.g., “Because it is an integral part of my life”), identified regulation (e.g., “Because it is a way to ensure long-term health benefits”), introjected regulation (e.g., “Because I would feel ashamed of myself if I did not do it”), external regulation (e.g., “Because others expect me to do it”), and amotivation (e.g., “I really do not know. I honestly feel it is a waste of time”). The scale has been validated in the Italian context [32]. For the purposes of the present study, a shortened version of the REBS was used, comprising three items for each motivational regulation, with the exception of amotivation, which included two items. The full wording of the retained items is reported in Appendix A. The decision to use a shortened version was a methodological choice and was guided by both theoretical and empirical considerations. Item selection was guided by the SDT framework, with the aim of preserving the representativeness of each regulatory style. In addition, selection was supported by prior psychometric evidence from validation studies, including item performance and factor loadings reported in previous work [32].

This approach was adopted to balance the reduction of questionnaire length and participant burden with the need to maintain adequate measurement quality. The psychometric adequacy of the shortened REBS in the present sample was subsequently evaluated through confirmatory factor analysis, reliability indices, convergent validity, and discriminant validity, as reported in the Section 3. All subscales demonstrated good internal reliability, with Cronbach’s alpha values of 0.89 for intrinsic motivation, 0.86 for integrated regulation, 0.85 for identified regulation, 0.82 for introjected regulation, 0.85 for external regulation, and 0.78 for amotivation.

Measures of perceived behavioral control (PBC) and behavioral intention were developed in accordance with standard guidelines [18] and based on instruments used in previous studies [15,16,17].

PBC was assessed using three items designed to capture participants’ perceived ability to eat healthily (e.g., “If I want to, I am confident that I can eat healthily”). Responses were provided on seven-point Likert-type scales ranging from 1 (strongly disagree) to 7 (strongly agree). Internal reliability in the present sample was satisfactory (Cronbach’s α = 0.76).

Behavioral intention was assessed using three items designed to capture participants’ intention to eat healthily (e.g., “I intend to eat healthily”). Responses were provided on seven-point Likert-type scales ranging from 1 (strongly disagree) to 7 (strongly agree). Internal reliability was good (Cronbach’s α = 0.82).

The level of adherence to the MD was assessed by the Questionnaire to Measure Mediterranean diet (QueMD) [33]. The QueMD is composed of 15 items, of which 9 explore the frequency of consumption of foods that are part of the MD, and 6 investigate foods that are not typically included in this dietary pattern. Each item has five possible response options, which vary according to the indicated consumption frequencies for each type of food [33]. The foods belonging to the MD include: whole-grain pasta or rice; all types of vegetables (raw or cooked); all types of fresh fruit and fresh fruit juices; whole-grain bread and substitutes; olive oil used for cooking or dressing; white and red wine (in moderate quantities); fish (fresh or frozen) or seafood; nuts (walnuts, almonds, hazelnuts); legumes (chickpeas, lentils, peas, beans). For each of these foods, one point was assigned when consumption exceeded a certain threshold, which varied from food to food, according to the guidelines of Gnagnarella et al. [33]. Among the foods not typically belonging to the MD are: milk and yogurt; butter, margarine, or cooking cream (10 g, equivalent to one tablespoon); red meats (beef, veal, pork) and processed meat products; white meats (chicken, turkey, rabbit) (100 g); carbonated and/or sugary drinks; homemade desserts, pastries, cookies, creams. Specifically, one point was assigned when the consumption of red meat did not exceed a certain threshold (maximum of 1–3 times per week). For the other items, no points were assigned, regardless of the consumption frequency [33]. These items mainly refer to non-Mediterranean and highly processed foods (e.g., manufactured sweets and sugary beverages) and are included to describe overall dietary patterns, rather than to contribute to the adherence score.

The total score ranges from 0 (minimal adherence to the MD) to 9 (maximum adherence to the MD). In young adults, this total score reflects alignment with core Mediterranean diet components rather than fine-grained variability in dietary intake. This level of aggregation is consistent with the aims of the present study.

### 2.4. Analysis

Data were initially analyzed by confirmatory factor analysis (CFA) in which motivational regulations, PBC, and intention were modeled as latent variables indicated by their respective items, to assess the construct validity of the study measures. Adherence to the MD was treated as an observed variable.

**Figure 1:**
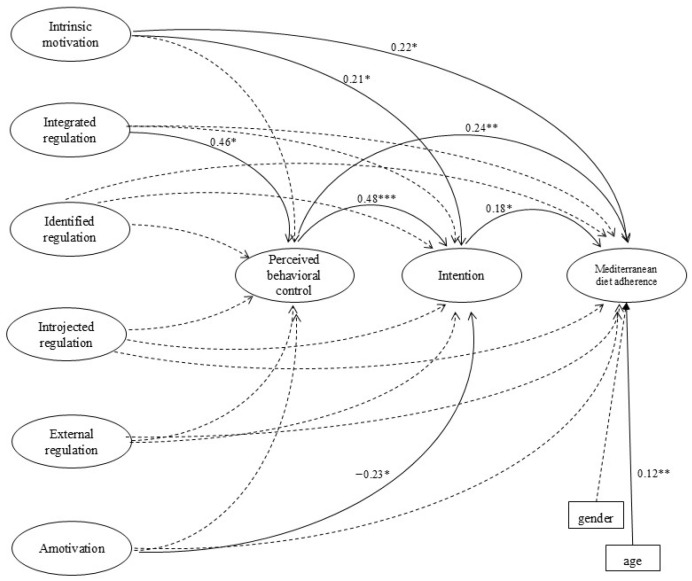
Structural Equation Model (SEM). The correlations among the different types of motivation were omitted for clarity. Note: Dotted lines represent insignificant pathways. * = *p* < 0.05; ** = *p* < 0.01; *** = *p* < 0.001.

Subsequently, the hypothesized relations among the different types of motivation, PBC, intention, and behavior were tested in a Structural Equation Model (SEM) (Figure 1). Age and gender were included as observed covariates in the regression model predicting adherence to the MD.

Structural equation modeling (SEM) was used to test whether the effects of different types of motivation on intention were mediated by PBC and whether the effects of PBC on behavior were mediated by intention. Finally, following Preacher and Hayes’ [34] procedure, hypothesized mediation effects were tested by calculating indirect effects and 95% confidence intervals using a bootstrapped resampling method with 5000 resamples. Mediation was confirmed by the presence of a statistically significant bootstrapped indirect effect (i.e., when the bootstrapped 95% confidence intervals [CIs] did not include zero).

Model fit was evaluated using the chi-square statistic (χ^2^), the comparative fit index (CFI), the Tucker–Lewis index (TLI), the root mean squared error of approximation (RMSEA), and the standardized root mean square residual (SRMR). Typically, a satisfactory model is indicated by non-significant χ^2^ values, CFI and TLI values ≥ 0.95, RMSEA values ≤ 0.06, and SRMR ≤ 0.08 [35].

All analyses were conducted using Mplus version 8. Maximum likelihood (ML) estimation was employed because the items were measured on Likert-type scales with more than five response categories, the data showed acceptable distributional properties, and the sample size exceeded 200. The dataset contained no missing values (0% missing data); therefore, no missing data handling procedures (e.g., full information maximum likelihood or imputation) were required.

## 3. Results

### 3.1. Descriptive Statistics

Three hundred and sixty-five participants completed the questionnaire (50.1% female; 21.74 years, SD = 5.86). Univariate analyses of variance of the effect of gender on behavior showed no statistically significant gender differences. Zero-order correlations between age and behavior were statistically significant (r = 0.11; *p* < 0.05). The daily consumption of the food items, assessed by the QueMD, is presented in Table 1. Descriptive statistics (means, standard deviations and observed ranges) for all key study variables, as well as their intercorrelations, are reported in Table 2. For each construct, scale scores were computed by averaging the corresponding item responses, with higher scores indicating higher levels of the measured construct.

### 3.2. Measurement Model

The measurement model was evaluated using confirmatory factor analysis (CFA). Results indicated an adequate fit to the data (χ^2^(223) = 417.996, *p* < 0.001, with a χ^2^/df ratio of 1.87; CFI = 0.961; TLI = 0.952; RMSEA = 0.049, 90% CI [0.041, 0.056], *p*-close = 0.604; SRMR = 0.065). All standardized factor loadings were statistically significant (*p* < 0.001), and generally exceeded the recommended cut-off value of 0.50. One item of PBC showed a slightly lower loading (λ = 0.46). The item was retained due to its theoretical relevance and because sensitivity analyses excluding this item yielded substantially unchanged results. The full set of standardized loadings is reported in Appendix A.

Factor correlations among latent constructs estimated from the CFA measurement model are reported in Appendix B. The correlations among the latent factors were consistent with theoretical expectations. In particular, strong positive correlations were observed among adjacent forms of motivational regulation (e.g., integrated and identified regulation, r = 0.82; integrated and intrinsic regulation, r = 0.67; identified and intrinsic regulation, r = 0.66), in line with the continuum-based conceptualization of motivation within SDT.

Convergent validity was assessed using Composite Reliability (CR) and Average Variance Extracted (AVE). Using the recommended thresholds (CR ≥ 0.70; AVE ≥ 0.50), all constructs demonstrated satisfactory internal consistency reliability and adequate convergent validity. Results of convergent validity for each construct are reported in Appendix C. Discriminant validity was assessed using both the Fornell–Larcker criterion and the Heterotrait–Monotrait ratio (HTMT). The square root of the AVE for each construct exceeded its correlations with all other constructs, satisfying the Fornell–Larcker criterion. In addition, all HTMT values ranged from 0.005 to 0.699, remaining well below the recommended threshold of 0.85. Together, these results provide evidence of adequate discriminant validity. Detailed results on discriminant validity for each construct are reported in Appendix D.

### 3.3. Model Testing

SEM analysis indicated an excellent fit of the hypothesized model to the data, with χ^2^(262) = 467.675, *p* < 0.0001, χ^2^/df = 1.785, RMSEA = 0.046 (90% CI [0.040, 0.053]), CFI = 0.959, TLI = 0.950, and SRMR = 0.047. The model, including age and sex as covariates, is depicted in Figure 1. Age showed a small but statistically significant positive association with adherence to the Mediterranean diet (β = 0.12, *p* < 0.01), whereas gender was not significantly associated with adherence (β = −0.08, *p* = 0.107). Adherence to MD behavior was positively and directly associated with intention (β = 0.18, *p* < 0.05), PBC (β = 0.24, *p* < 0.01) and intrinsic motivation (β = 0.22, *p* < 0.05), while no significant direct associations were observed for the other types of regulations or amotivation. Intention showed significant positive associations with PBC (β = 0.48, *p* < 0.001) and intrinsic motivation (β = 0.21, *p* < 0.05) and was negatively associated with amotivation (β = −0.23, *p* < 0.05), whereas other forms of motivation did not show significant associations. PBC was positively associated with integrated regulation (β = 0.46, *p* < 0.01), while other motivational variables had no significant direct associations. The inter-correlations among different types of motivation reflect the complex structure of motivational regulation. Overall, the model accounted for 40% of the variance in intention and 16% of the variance in adherence to MD behavior. The proportion of explained variance in adherence should be interpreted as modest, yet consistent with what is typically observed for complex health-related behaviors, underscoring the role of additional contextual and structural determinants.

The indirect effects analysis (Table 3) identified some significant mediated effects in the structural equation model of adherence to the MD.

PBC showed a significant indirect association with adherence behavior via intention. Integrated regulation also showed a significant indirect association with intention via PBC. No significant indirect effects were found for intrinsic motivation, identified regulation, introjected regulation, external regulation or amotivation via PBC on intention. Overall, adherence to the MD was associated both directly and indirectly with PBC and integrated regulation, with intention emerging as a key mediating variable. Notably, mediation through PBC accounted for a meaningful proportion of the total associations with intention and adherence to the MD.

## 4. Discussion

The present study aimed to investigate the adoption of the MD among young adults by integrating motivational constructs from SDT with PBC and intention derived from the TPB. Overall, the findings support the adoption of this integrated framework for the study of adherence to the MD, extending previous research that has mainly focused on specific dietary behaviors or on adult samples [15,20].

A first pattern of results emerges with respect to adherence to the MD. Consistent with TPB, intention emerged as a factor directly associated with dietary behavior. In addition, PBC showed a direct association with adherence, as well as an indirect association through intention. This suggests that when young adults form a concrete intention to adopt a specific behavior, this intention is often grounded in their perception of having the necessary skills, time, and access to resources to enact that behavior. In the context of MD adherence, this may involve having basic cooking skills for simple vegetable-based and legume-based meals, access to well-stocked food outlets, or the availability of local markets.

Notably, intrinsic motivation showed a direct association with adherence to the Mediterranean diet, indicating that, beyond cognitive determinants, enjoyment and genuine interest in healthy eating may contribute to sustaining actual dietary behavior even when intention and perceived behavioral control are taken into account.

In this respect, our integrated model—explaining 40% of the variance in intention and 16% of the variance in adherence to the MD—largely converges with previous studies by Canova [20], Caso [21] and Girelli [15,36], confirming the central role of the PBC as a mediator between autonomous forms of motivation and behavioral intention. However, it also extends these findings by showing a significant direct association of intrinsic motivation with dietary adherence, even when controlling for intention, a pattern that has not consistently emerged in prior research, particularly in studies focusing on healthy eating in general.

At the same time, the proportion of variance explained should be interpreted as modest. This suggests that, while motivational and social–cognitive variables play a meaningful role in explaining adherence to the MD, a substantial proportion of variance remains attributable to contextual and structural factors not included in the present model. These may include the food environment and access, availability of healthy options on campus, cooking resources, time constraints, affordability, the social environment, and culturally embedded eating patterns, which can either facilitate or constrain individuals’ ability to translate motivational intentions into actual behavior.

A second pattern concerns intention to adopt a healthy diet consistent with the Mediterranean dietary pattern. PBC showed the strongest association with intention, confirming its central role in shaping young adults’ readiness to adhere to the MD. Among motivational variables, intrinsic motivation showed a positive association with intention, whereas amotivation showed a negative one. Other forms of regulation did not show significant direct effects. This configuration suggests that intention is supported when individuals both feel capable of managing dietary choices and experience interest or enjoyment in healthy eating, whereas a lack of motivation is associated with weaker intention. This finding is in line with previous research showing associations between PBC and more autonomous forms of motivation as conceptualized by SDT [15,20,37]. Prior studies suggest that individuals with self-determined motives are more likely to develop stronger beliefs about the availability of resources needed to enact a behavior. One possible explanation is that autonomous motivation fosters the pursuit of personally meaningful goals and enhances perceived competence in managing the required actions.

A third pattern emerges from the analysis of indirect effects and concerns the role of PBC as a linking mechanism between motivation and intention. No significant indirect effects were observed for the other forms of motivation.

This might suggest that when healthy eating becomes part of one’s self-concept, individuals feel more competent in performing the activities required to follow the MD, from grocery shopping to meal planning. The integration of dietary values may therefore enhance PBC, thereby supporting the formation of intention and facilitating behavioral enactment.

This pattern is in line with integrated SDT–TPB models, suggesting that motivational regulations may influence intention indirectly by shaping individuals’ perceptions of control and competence [15,20,37]. The present study contributes to this line of research by showing that such indirect pathways are observable when focusing on adherence to the MD among young adults. In particular, the findings suggest that more autonomous forms of motivation may support intention primarily through PBC, whereas externally regulated motives may undermine it. This pattern extends previous evidence obtained on specific eating behaviors by showing similar mechanisms at work for a broader dietary pattern.

Consistent with SDT, some motivational regulations that are theoretically adjacent along the motivational continuum (e.g., integrated and identified regulation) were strongly correlated. This pattern is in line with previous research [38] and reflects the conceptual proximity of these forms of autonomous motivation.

From a modeling perspective, such overlap raises the possibility that a more parsimonious representation based on higher-order factors (e.g., autonomous vs. controlled motivation) or composite scores could be considered. However, in the present study, we retained the more fine-grained structure in order to preserve conceptual specificity and to examine potentially distinct indirect pathways associated with different regulatory styles.

Future research could explicitly compare alternative model specifications to further evaluate the relative advantages of fine-grained versus higher-order motivational representations in the prediction of dietary intentions and adherence.

Notably, identified and introjected regulation did not show significant direct or indirect effects on intention or adherence to the Mediterranean diet. Although these forms of motivation are partially internalized, they may be less effective in sustaining adherence to a complex and demanding dietary pattern such as the MD during emerging adulthood. During this developmental phase, eating behaviors may be more strongly driven by intrinsic enjoyment and by values that are fully integrated into one’s identity, rather than by motives based on recognizing the benefits of the behavior or on guilt avoidance. Identified and introjected regulation may instead play a more relevant role in the initiation of specific or short-term dietary changes, rather than in the maintenance of a broader dietary pattern requiring sustained effort and planning.

Taken together, these findings are consistent with existing literature on the integration of SDT and TPB, while also extending it in two main ways. Specifically, this study contributes to prior research by focusing on adherence to the Mediterranean diet as a comprehensive dietary pattern rather than on isolated eating behaviors, and by examining these processes in a sample of young adults, a developmental stage that has received comparatively limited attention in this domain.

The present findings should be interpreted considering several limitations. First, the cross-sectional design does not allow conclusions about the directionality of the relationships among motivational regulations, social–cognitive variables, and adherence to the MD. Future studies adopting longitudinal or experimental designs would be valuable in clarifying how these processes evolve over time.

Second, all variables were assessed through self-report measures, which may be subject to bias, including inaccuracies in self-reported dietary intake and social desirability effects. Moreover, because motivational regulations, PBC, intention, and adherence to the MD were assessed using self-report measures and collected within a single survey session, the possibility of common-method bias cannot be ruled out. In such conditions, shared response tendencies and consistency motives may increase common-method variance, potentially inflating the observed associations among constructs. Although this limitation is common in research on health-related behaviors, it should be considered when interpreting the magnitude of the reported effects. Future research could address this issue by incorporating objective indicators of dietary adherence or health status (e.g., BMI or biomarkers), as well as by adopting multi-method or multi-source designs, temporal separation of measurements, or other design-based strategies aimed at reducing common-method variance.

Third, the sample consisted primarily of young adults, many of whom were university students. Although this focus is consistent with the aims of the study and targets a relatively underexplored developmental stage, caution is warranted when generalizing the findings to other populations. In addition, the assessment of sociodemographic and contextual factors was limited to basic indicators (e.g., age, gender, and student status), while other relevant variables—such as socioeconomic conditions, living arrangements, employment status, and broader contextual characteristics that may shape perceived behavioral control and dietary adherence during emerging adulthood—were not assessed.

Finally, the limited representativeness of the sample highlights the need to replicate the proposed model in more diverse groups of young adults, including individuals with different educational and occupational backgrounds and from a wider range of sociocultural contexts.

Despite these limitations, the present findings provide several implications for educational and healthcare practice. In nutritional communication and counseling, autonomy-supportive approaches may be particularly effective. For instance, short-term interventions that foster a personal connection to healthy eating, support a sense of choice, and enhance awareness of personal values could be integrated into university health services or primary care settings.

In addition, interventions aimed at strengthening perceived behavioral control could focus on enhancing individuals’ sense of mastery over their dietary choices, for example, through workshops on meal planning, guidance for making healthier choices in cafeterias or restaurants, or introductory activities related to simple Mediterranean cuisine.

Finally, assessing young adults’ motivational profiles may help tailor interventions more effectively, distinguishing between individuals who may benefit most from strategies designed to strengthen autonomous motivation and those who may require support to reduce amotivation.

## 5. Conclusions

The present study examined adherence to the MD among young adults using an integrated framework combining SDT and selected socio-cognitive constructs from the TPB—namely PBC and behavioral intention. The findings indicate that adherence is associated with both intention and PBC and that motivational regulations contribute in differentiated ways. In particular, intrinsic motivation shows a direct association with adherence to the MD, whereas integrated regulation appears to be related to dietary behavior mainly through its associations with PBC and intention. By focusing on adherence to the MD as a comprehensive dietary pattern and on a population of young adults, this study extends previous research, which has largely examined these processes in relation to specific eating behaviors or adult samples. Overall, the results support the applicability of established motivational and social–cognitive frameworks to the study of dietary adherence during emerging adulthood, highlighting the value of integrating motivational and socio-cognitive perspectives to better understand and promote healthy eating behaviors.

## Figures and Tables

**Table 1:** Daily consumption of food items assessed by the QueMD.

Food Items	QueMD (Frequency of Consumption)					
	Reference portions	Never or seldom	<1 per day	1 per day	2 per day	≥3 per day
1. Wholegrain pasta or rice	80 g	26.9%	18.5%	45.9%	6.0%	2.7%
2. Vegetables, all types (raw and cooked)	200 g 80 g (salad)	7.1%	21.2%	36.1%	31.0%	4.6%
3. Fruits, all types (fresh and fresh juices)	150 g	12.0%	27.2%	31.5%	23.1%	6.3%
4. Milk and yoghurt	125 g	16.0%	23.9%	39.1%	13.9%	7.1%
		Never or seldom	<1 per day	1–2 per day	3–4 per day	≥5 per day
5. Wholegrain bread and substitutes	50 g (1–2 slices)	29.9%	26.1%	36.7%	5.4%	1.9%
6. Olive oil to cook and to dress	10 mL (1 spoon)	5.4%	15.5%	65.2%	9.8%	4.1%
7. Butter, margarine or cooking cream	10 g (1 spoon)	43.2%	39.1%	14.4%	2.2%	1.1%
8. Wine (white and red)	125 mL (1 glass)	67.4%	21.7%	7.1%	1.9%	1.9%
		Never or seldom	<1 per week	1–3 per week	4–6 per week	≥7 per week
9. Red meat (beef, veal, pork), meat products	100 g (raw meat) 50 g (meat products)	4.9%	18.8%	64.9%	9.5%	1.9%
10. White meat (chicken, turkey, rabbit)	100 g	3.5%	14.1%	63.9%	16.8%	1.6%
11. Carbonated and/or sugared/sweetened beverages	200 mL (1 glass)	21.7%	35.6%	28.0%	7.9%	6.8%
12. Manufactured sweets, pastries, biscuits, creams.	100 g	20.4%	39.4%	33.2%	3.8%	3.3%
		Never or seldom	<1 per week	1 per week	2–3 per week	≥4 per week
13. Fish (fresh or frozen) or seafood	150 g (fish) 50 g (fish products)	13.3%	22.8%	35.3%	25.3%	3.3%
14. Dried fruits (nuts, almonds, hazelnuts)	30 g (1 fist)	19.8%	29.3%	21.7%	20.7%	8.4%
15. Pulses (chickpeas, lentils, peas, beans)	50 g (dried) 150 g (canned/raw)	7.6%	13.3%	26.4%	44.8%	7.9%

**Table 2:** Mean, standard deviations, ranges and intercorrelations for the key variables of the study.

	M	SD	Ranges	1	2	3	4	5	6	7	8	9
(1) Intrinsic motivation	3.44	1.74	1–7	-								
(2) Integrated regulation	3.92	1.67	1–7	0.65 **	-							
(3) Identified regulation	4.76	1.61	1–7	0.62 **	0.72 **	-						
(4) Introjected regulation	2.89	1.69	1–7	0.24 **	0.27 *	0.31 **	-					
(5) External regulation	2.22	1.51	1–7	0.23 **	0.16 **	0.12 *	0.54 **	-				
(6) Amotivation	2.10	1.42	1–7	0.19 **	0.05	0.01	0.40 **	0.58 **	-			
(7) PBC	4.34	1.60	1–7	0.32 **	0.38 **	0.36 **	0.13 *	−0.02	−0.00	-		
(8) Intention	3.14	1.63	1–7	0.36 **	0.37 **	0.29 **	0.16 **	0.10	0.03	0.45 **	-	
(9) MD	3.59	1.69	0–9	0.28 **	0.27 **	0.27 **	0.04	0.02	0.03	0.27 **	0.30 **	-

PBC = Perceived behavioral control; MD = Mediterranean Diet. Note. * = *p* < 0.05. ** = *p* < 0.01.

**Table 3:** Standardized path coefficients for mediated effects in structural equation model of adherence to the MD.

Paths	Mediator	Direct Effect		Indirect Effect		Total Effect		Mediation
		Std. Estimation	95% CI Low/High	Std. Estimation	95% CI Low/High	Std. Estimation	95% CI Low/High	
PBC → MD	Intention	0.243 *	0.086/0.396	0.089 *	0.018/0.168	0.331 ***	0.205/0.451	Yes
INTRI → INT	PBC	0.211 *	0.010/0.412	0.086	−0.011/0.193	0.297 **	0.100/0.506	No
INTE → INT	PBC	0.123	−0.207/0.440	0.224 **	0.067/0.422	0.347 *	0.050/0.654	Yes
IDE → INT	PBC	−0.197	−0.559/0.117	−0.044	−0.253/0.149	−0.240	−0.606/0.040	No
INTRO → INT	PBC	0.123	−0.107/0.372	0.026	−0.081/0.143	0.149	−0.068/0.406	No
EXT → INT	PBC	0.188	−0.022/0.450	−0.097	−0.258/0.024	0.091	−0.139/0.360	No
AMO → INT	PBC	−0.234 *	−0.487/−0.075	−0.053	−0.082/0.198	−0.181	−0.465/0.008	No

PBC = Perceived behavioral control; MD = Mediterranean Diet; INTRI = Intrinsic motivation; INTE = Integrated regulation; IDE = Identified regulation; INTRO = Introjected regulation; EXT = External regulation; AMO = Amotivation; INT = Intention Note. 95% bias-corrected bootstrap confidence intervals were calculated using 5000 resamples. * = *p* < 0.05. ** = *p* < 0.01. *** = *p* < 0.001.

## Data Availability

The raw data supporting the conclusions of this article will be made available by the authors on request.

## Abbreviations

The following abbreviations are used in this manuscript:

MD	Mediterranean Diet
PBC	Perceived Behavioral Control
SDT	Self-Determination Theory
TPB	Theory of Planned Behavior
WHO	World Health Organization

## Appendix A

**Table A1:** Item wording and standardized factor loadings of the measurement model.

	Item n.	Item Wording	St. Loadings
Intrinsic motivation	Item 1	Because it is fun	0.863
	Item 2	Because I enjoy finding new ways to prepare healthy meals	0.845
	Item 3	Because I enjoy preparing healthy meals	0.881
Integrated regulation	Item 1	Because it is an integral part of my life	0.829
	Item 2	Because eating healthy is part of the way I have chosen to live my life	0.816
	Item 3	Because eating healthy is consistent with other important aspects of my life	0.815
Identified regulation	Item 1	Because I believe that eating healthy will make me feel better	0.793
	Item 2	Because I think it is a good thing to try to regulate my eating behaviors	0.815
	Item 3	Because it is a way to ensure long-term health benefits	0.821
Introjected regulation	Item 1	Because I feel that I must absolutely be thin	0.742
	Item 2	Because I would feel ashamed of myself if I did not do it	0.786
	Item 3	Because I would feel humiliated if I were unable to control my eating behaviors	0.788
External regulation	Item 1	Because people close to me insist that I eat healthy	0.802
	Item 2	Because people around me pressure me to eat healthy	0.824
	Item 3	Because others expect me to do it	0.805
Amotivation	Item 1	I really do not know. I honestly feel it is a waste of time	0.78
	Item 2	I really cannot see what I would gain from eating healthy	0.82
Perceived behavioral control	Item 1	I think that eating healthy would be very easy for me	0.801
	Item 2	Whether I succeed in eating healthy depends mostly on me	0.461
	Item 3	If I want to, I am confident that I can eat healthy	0.701
Intention	Item 1	I intend to eat healthy	0.908
	Item 2	I plan to eat healthy	0.904
	Item 3	I expect to eat healthy	0.895

## Appendix B

**Table A2:** Factor correlations among latent constructs estimated from the CFA.

Latent Construct	(1)	(2)	(3)	(4)	(5)	(6)
(1) Intrinsic motivation	-					
(2) Integrated regulation	0.67 ***	-				
(3) Identified regulation	0.66 ***	0.82 ***	-			
(4) Introjected regulation	0.23 ***	0.25 ***	0.30 ***	-		
(5) External regulation	0.23 ***	0.13	0.15 *	0.68 ***	-	
(6) Amotivation	0.13 *	−0.02	−0.11	0.49 ***	0.68 ***	-

* *p* < 0.05. *** *p* < 0.001.

## Appendix C

**Table A3:** Composite Reliability (CR) and Average Variance Extracted (AVE) for each latent construct of the study.

Latent Constructs	CR	AVE
Intrinsic motivation	0.90	0.76
Integrated regulation	0.87	0.68
Identified regulation	0.87	0.66
Introjected regulation	0.85	0.61
External regulation	0.88	0.65
Amotivation	0.81	0.65
Perceived behavioral control	0.82	0.73
Intention	0.93	0.81

## Appendix D

**Table A4:** Discriminant validity assessment using the Fornell–Larcker criterion.

	INTRI	INTE	IDE	INTRO	EXT	AMO	INT	PBC
INTRI	0.872	0.587	0.578	0.198	0.197	0.105	0.367	0.312
INTE	0.587	0.825	0.699	0.195	0.099	−0.033	0.360	0.363
IDE	0.578	0.699	0.812	0.251	0.125	−0.091	0.282	0.346
INTRO	0.198	0.195	0.251	0.781	0.562	0.399	0.182	0.110
EXT	0.197	0.099	0.125	0.562	0.806	0.556	0.123	0.006
AMO	0.105	−0.033	−0.091	0.399	0.556	0.806	0.008	−0.005
INT	0.367	0.360	0.282	0.182	0.123	0.008	0.854	0.428
PBC	0.312	0.363	0.346	0.110	0.006	−0.005	0.428	0.900

Note. Diagonal elements represent the square root of AVE; off-diagonal elements represent inter-construct correlations.

**Table A5:** Discriminant validity assessment using the Heterotrait–Monotrait ratio (HTMT).

	INTRI	INTE	IDE	INTRO	EXT	AMO	INT	PBC
INTRI	1.000	0.587	0.578	0.198	0.197	0.105	0.367	0.312
INTE	0.587	1.000	0.699	0.195	0.099	0.033	0.360	0.363
IDE	0.578	0.699	1.000	0.251	0.125	0.091	0.282	0.346
INTRO	0.198	0.195	0.251	1.000	0.562	0.399	0.182	0.110
EXT	0.197	0.099	0.125	0.562	1.000	0.556	0.123	0.006
AMO	0.105	0.033	0.091	0.399	0.556	1.000	0.008	0.005
INT	0.367	0.360	0.282	0.182	0.123	0.008	1.000	0.428
PBC	0.312	0.363	0.346	0.110	0.006	0.005	0.428	1.000

Note. HTMT values below 0.85 indicate adequate discriminant validity.
